# Effects of Extended-Release Polyhexanide on Inflammatory Cytokine Expression and Disease Severity Following Staphylococcus Aureus Exposure in a Murine Dermatitis Model

**DOI:** 10.3390/life16081369

**Published:** 2026-08-20

**Authors:** Bradley Burnam, Stephen D. Bresnick

**Affiliations:** 1 Turn Therapeutics, Westlake Village, CA 91362, USA; 2 Independent Researcher, Los Angeles, CA 91346, USA

**Keywords:** epithelial biology, inflammatory signaling, innate immunity, host–microbe interactions, skin biology, cytokines, polyhexanide

## Abstract

Background: The skin functions as an active immunologic interface in which microbial exposure triggers epithelial stress responses and downstream inflammatory signaling. *Staphylococcus aureus* is a potent inducer of cutaneous inflammation and has been shown to stimulate expression of IL-36 cytokines, which function as upstream amplifiers of immune activation. The extent to which modulation of the cutaneous microenvironment influences these signaling pathways remains incompletely understood. Objective: To evaluate the effects of cutaneous microenvironment modulation using extended-release polyhexanide formulation GX-03 on a murine model of *Staphylococcus aureus*-induced dermatitis. Specifically, we evaluated whether pretreatment with GX-03 was associated with changes in IL-36-mediated epithelial stress signaling, selective downstream cytokine expression, and clinical disease severity following standardized epicutaneous *Staphylococcus aureus* exposure. Methods: Forty female C57BL/6 mice underwent epicutaneous *Staphylococcus aureus* challenge following either no pretreatment or four days of topical GX-03 pretreatment. Animals were randomized equally into two treatment groups. Following exclusion of four animals because of premature loss of the occlusive dressing, analyses were performed on 18 animals per group. Disease severity was assessed using a validated Investigator Global Assessment for Atopic Dermatitis (vIGA-AD) scoring system. Skin tissue cytokine expression was quantified by Western blot analysis. Results: GX-03 pretreatment significantly reduced disease severity compared with untreated controls (mean vIGA-AD 0.83 vs. 2.44, *p* < 0.001). Significant reductions were observed in IL-36α (49.9%), IL-36γ (50.9%), IL-31 (67.7%), and IL-4 (16.7%). IL-13 expression was unchanged (*p* = 0.988). Conclusions: Pretreatment with extended-release polyhexanide (GX-03) reduced disease severity and was associated with decreased expression of IL-36α, IL-36γ, IL-31, and IL-4 following epicutaneous *Staphylococcus aureus* exposure, while IL-13 expression remained unchanged. These findings suggest that modulation of the cutaneous microenvironment is associated with selective alterations in inflammatory cytokine expression following microbial challenge. Additional studies incorporating microbiologic, histologic, and mechanistic endpoints are needed to further define the biological mechanisms responsible for these observations.

## 1. Introduction

The skin serves as the primary interface between the host and the external environment and plays an important role in regulating inflammatory responses to microbial exposure. In addition to its barrier function, the epidermis actively participates in immune signaling through the production of cytokines and other inflammatory mediators. Disruption of epidermal homeostasis by microbial challenge, barrier injury, or environmental stress can initiate signaling pathways that contribute to cutaneous inflammation [1,2,3].

Among microorganisms implicated in inflammatory skin disease, *Staphylococcus aureus* has received considerable attention because of its ability to induce epithelial activation and cytokine production [4,5]. Experimental murine models have demonstrated that epicutaneous *S. aureus* exposure produces reproducible inflammatory skin disease characterized by erythema, scaling, epidermal hyperplasia, and immune activation [3,4]. These models have provided valuable insight into mechanisms underlying host–microbe interactions at the skin barrier.

The IL-36 cytokine family has emerged as an important component of the epithelial response to microbial challenge. IL-36α and IL-36γ are rapidly induced following epithelial injury and exposure to microbial products, where they function as upstream amplifiers of inflammatory signaling [4,5,6,7]. Experimental studies have demonstrated that IL-36 signaling contributes to *S. aureus*-induced skin inflammation and promotes activation of multiple downstream inflammatory pathways [3,4]. Consequently, IL-36 cytokines have become useful markers of epithelial stress-associated inflammatory activation.

Polyhexanide (polyhexamethylene biguanide; PHMB) is a broad-spectrum agent with established activity against Gram-positive and Gram-negative microorganisms, including *Staphylococcus aureus* [8]. Previous studies have demonstrated reductions in bacterial viability and biofilm formation following controlled polyhexanide exposure, suggesting that alteration of the local microbial environment may influence inflammatory responses associated with microbial challenge [9,10]. GX-03 is a proprietary extended-release topical formulation designed to provide sustained topical delivery of polyhexanide without sensitization. In a previous study utilizing the same Staphylococcus aureus-induced murine dermatitis model, GX-03 significantly reduced clinical dermatitis severity following microbial challenge [11]. The present investigation extends those findings by examining whether pretreatment with GX-03 was associated with changes in epithelial stress-associated cytokine expression. Specifically, we evaluated IL-36α and IL-36γ, together with the downstream inflammatory mediators IL-31, IL-4, and IL-13, to better define the relationship between microbial challenge, epithelial activation, and inflammatory signaling following pretreatment with GX-03.

The present study was undertaken to evaluate cytokine responses following epicutaneous *Staphylococcus aureus* exposure and to determine whether pretreatment with GX-03 was associated with alterations in epithelial stress-associated inflammatory signaling. We hypothesized that pretreatment with GX-03 would attenuate expression of selected epithelial stress-associated cytokines following a standardized bacterial challenge while reducing the severity of the resulting inflammatory skin disease.

## 2. Materials and Methods

### 2.1. Study Design

**Figure 1:**
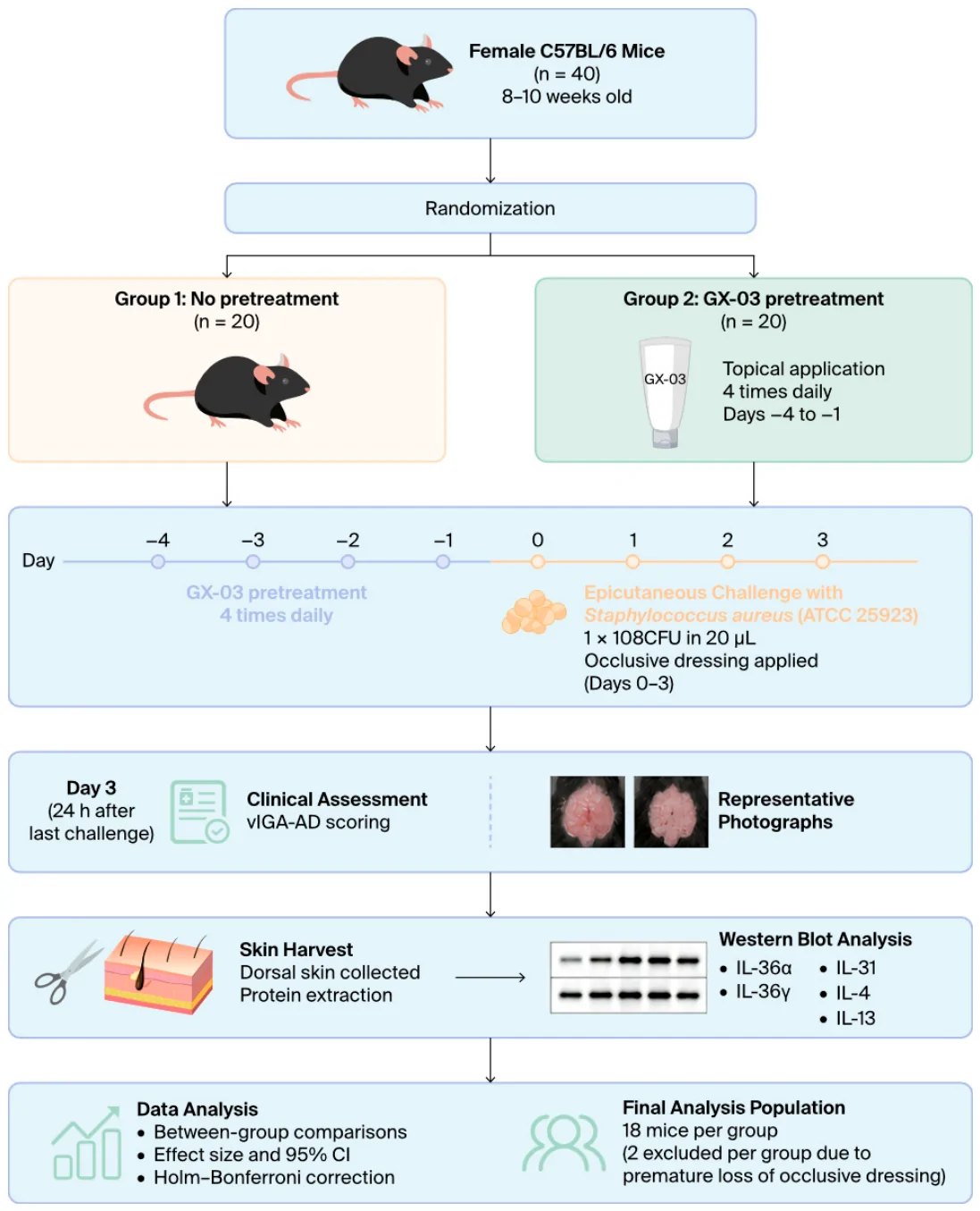
Graphical workflow of the study.

Animal studies were conducted at Altogen Labs (Austin, TX, USA) under IACUC-approved protocol LC04785. The individual mouse was considered the experimental unit for all analyses. Forty female C57BL/6 mice aged 8–12 weeks were obtained from Envigo Laboratories (St. Louis, MO, USA). Animals were housed in individually ventilated cages under standardized temperature- and humidity-controlled conditions with a 12 h light/dark cycle and ad libitum access to food and water. Following a one-week acclimatization period, animals were randomized equally into two experimental groups (20 animals per group). Four animals (two per group) were excluded after premature loss of the occlusive dressing during bacterial exposure, resulting in a final analysis of 18 animals per group. Animals were monitored daily for general health and signs of distress in accordance with the approved IACUC protocol. A graphical workflow for the study is shown in Figure 1.

### 2.2. Gx-03 and Extended-Release Polyhexanide

GX-03 is a proprietary extended-release topical formulation containing 0.5% w/w polyhexanide dispersed within a sustained-release lipid matrix. The formulation consists of a dispersion-based system in which polyhexanide is suspended as finely dispersed microdroplets (mean droplet size approximately 1.3 μm), facilitating controlled release of the water-soluble active agent following application to the skin. Upon contact with the skin at physioP{rlogical temperature, the formulation is designed to provide sustained local delivery of polyhexanide within the epidermal microenvironment. The present study evaluated the complete proprietary formulation as the investigational product.

The four-times-daily pretreatment schedule was selected during protocol development to establish consistent tissue exposure before bacterial challenge and to ensure reproducible pretreatment conditions within the experimental model.

Animals were assigned to one of two treatment groups:

Group 1: No pretreatment.

Group 2: GX-03 pretreatment administered topically four times daily for four consecutive days.

### 2.3. Pretreatment and Bacterial Challenge

Pretreatment was initiated in Group 2 by applying GX-03 four times daily for four consecutive days. No evidence of local irritation, xerosis, erythema, barrier disruption, or other adverse cutaneous findings was observed during the pretreatment period before bacterial challenge. Following either no pretreatment (Group 1) or GX-03 pretreatment (Group 2), all animals underwent standardized epicutaneous *Staphylococcus aureus* exposure.

The bacterial inoculum was prepared from *Staphylococcus aureus* (ATCC 25923). Bacteria were cultured to mid-logarithmic phase, washed, resuspended in sterile phosphate-buffered saline (PBS), standardized to 1 × 10^8^ colony-forming units (CFU)/100 μL by optical density measurement, and verified by overnight culture on tryptic soy agar before application. A 100 μL bacterial suspension was applied to a sterile 1 × 1 cm gauze pad, which was secured to the dorsal skin using an occlusive Tegaderm dressing and adhesive bandage for three consecutive days. Quantitative bacterial recovery from the skin following inoculation was not performed because the primary objective of the study was to evaluate the host inflammatory response following a standardized bacterial challenge rather than bacterial colonization or clearance.

### 2.4. Assessment of Disease Severity

Disease severity was evaluated by blinded study personnel following three days of *Staphylococcus aureus* exposure using the validated Investigator Global Assessment for Atopic Dermatitis (vIGA-AD) [12]. This standardized dermatitis severity scale ranges from 0 to 4 and assesses erythema, scaling, crusting, excoriation, and skin thickening.

Representative clinical photographs were obtained at the completion of the study to document the gross phenotypic differences between untreated and GX-03-pretreated animals.

### 2.5. Tissue Collection and Cytokine Analysis

Skin tissue was harvested following study completion, washed in phosphate-buffered saline (PBS), homogenized in lysis buffer containing protease inhibitor, and centrifuged to obtain protein lysates. Total protein concentration was determined using a Bradford assay (Bio-Rad, Hercules, CA, USA), and all samples were normalized to 1 mg/mL before analysis. Equal amounts of total protein (10 μg per lane) were separated on 4–15% precast SDS-polyacrylamide gels (Bio-Rad) before transfer to polyvinylidene difluoride (PVDF) membranes. Membranes were blocked with 5% non-fat milk in PBS and incubated with primary antibodies against IL-36α, IL-36γ, IL-31, IL-4, and IL-13, followed by species-appropriate secondary antibodies. Densitometric analysis was performed using GelAnalyzer 23.1.1 software. Protein normalization was achieved by Bradford assay and equal protein loading rather than housekeeping protein expression.

### 2.6. Statistical Analysis

Clinical scores and cytokine expression data were analyzed using GraphPad Prism (version 11.0.2). Continuous variables are presented as mean ± standard deviation. Prior to inferential analysis, data distribution was assessed using the Shapiro–Wilk test, and homogeneity of variance was evaluated using Levene’s test. Between-group comparisons were performed using two-sided independent-samples Student’s *t*-tests when assumptions of equal variance were met and Welch’s *t*-tests when variances were unequal. Mean differences, 95% confidence intervals, and Cohen’s d effect sizes were calculated. Holm-adjusted *p* values were calculated to account for multiple comparisons. Mann–Whitney U tests were performed as sensitivity analyses where appropriate. Statistical significance was defined as a two-sided adjusted *p* < 0.05.

## 3. Results

### 3.1. Clinical Disease Severity

**Figure 2:**
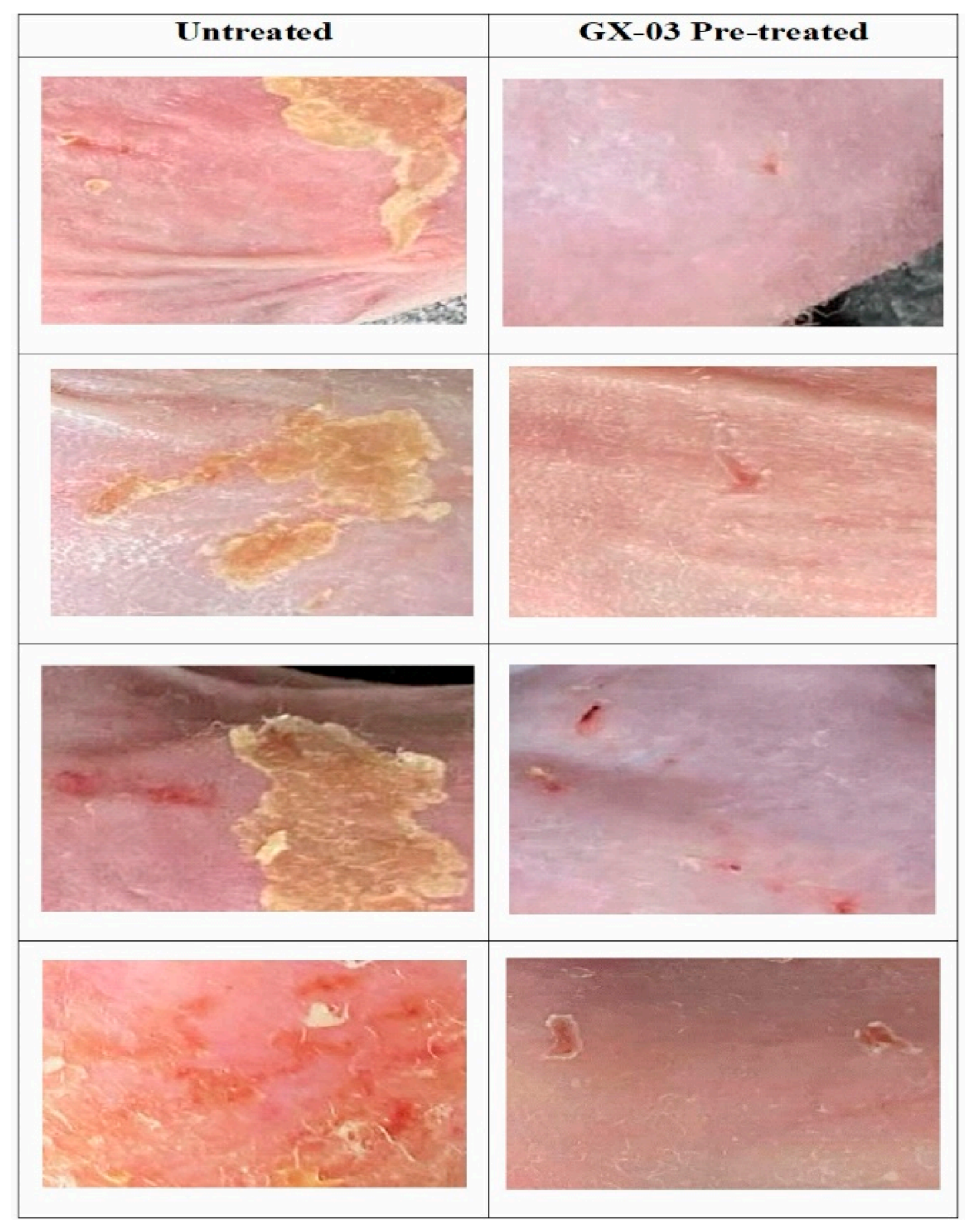
Untreated and GX-03 Pretreated Animals Following Epicutaneous *Staphylococcus aureus* exposure.

Standardized epicutaneous *Staphylococcus aureus* exposure produced clinically apparent dermatitis in untreated animals characterized by erythema, scaling, crusting, and epidermal irritation. Animals pretreated with GX-03 demonstrated less severe dermatitis. Representative clinical photographs of untreated and GX-03-pretreated animals are shown in Figure 2.

**Figure 3:**
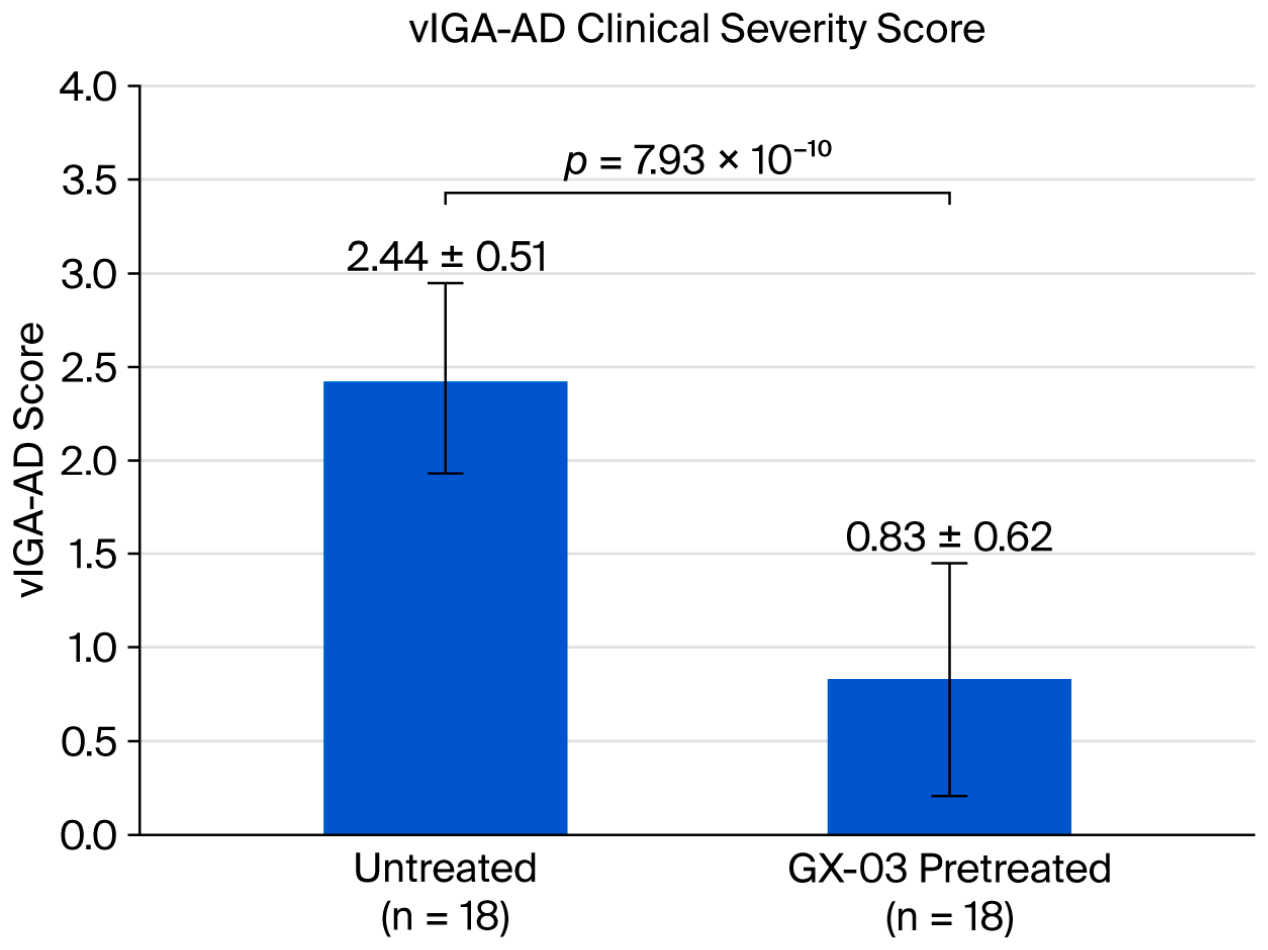
vIGA-AD Scores for Untreated and GX-03 Pretreated Animals.

All animals tolerated the four-day pretreatment period without evidence of local irritation, xerosis, erythema, or barrier disruption before bacterial challenge. Following exclusion of four animals because of premature loss of the occlusive dressing (two animals per group), clinical and cytokine analyses were performed on 36 animals (18 per group). Pretreatment with GX-03 was associated with a marked reduction in clinical disease severity after microbial challenge. Mean vIGA-AD scores decreased from 2.44 ± 0.51 in untreated animals to 0.83 ± 0.62 following GX-03 pretreatment, representing a 66.0% reduction in disease severity (exact *p* = 7.93 × 10^−10^). A graphical representation of vIGA-AD scores is shown in Figure 3.

### 3.2. Il-36 Expression

Western blot analysis demonstrated robust induction of the epithelial stress-associated cytokines IL-36α and IL-36γ following standardized epicutaneous *Staphylococcus aureus* exposure (Figures S1 and S2). Pretreatment with GX-03 was associated with substantial reductions in expression of both cytokines (Table 1).

**Figure 4:**
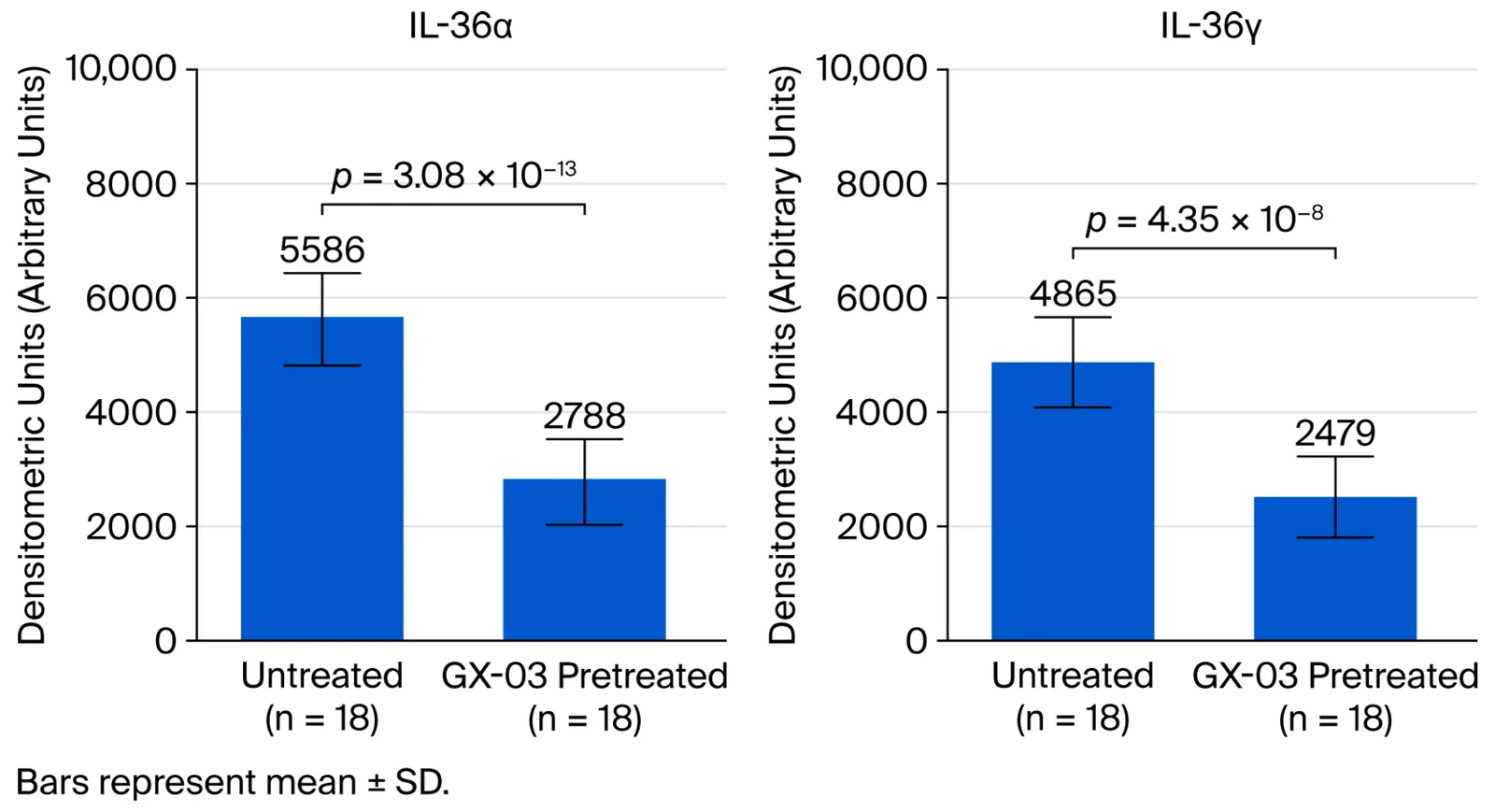
Densitometric Units (AU) for IL-36α and IL-36γ in Untreated and GX-03 Pretreated animals.

IL-36α expression decreased from 5586 ± 701 AU in untreated animals to 2788 ± 761 AU following GX-03 pretreatment, representing a 49.9% reduction (mean difference, −2798 AU; 95% confidence interval [CI], −3294 to −2302 AU; Cohen’s d = 3.82). Statistical comparison using a two-sided Student’s *t*-test demonstrated a highly significant reduction (exact *p* = 3.08 × 10^−13^), which remained significant following Holm adjustment for multiple comparisons. Similarly, IL-36γ expression decreased from 4865 ± 401 AU in untreated animals to 2479 ± 1387 AU following GX-03 pretreatment, corresponding to a 50.9% reduction. Welch’s *t*-test demonstrated a highly significant reduction (exact *p* = 8.88 × 10^−7^), which remained significant following Holm adjustment. Figure 4 graphically displays IL-36α and IL-36γ densitometric units for untreated and GX-03 pre-treated animals.

### 3.3. Downstream Cytokine Responses (IL-4, IL-31, IL-13)

**Figure 5:**
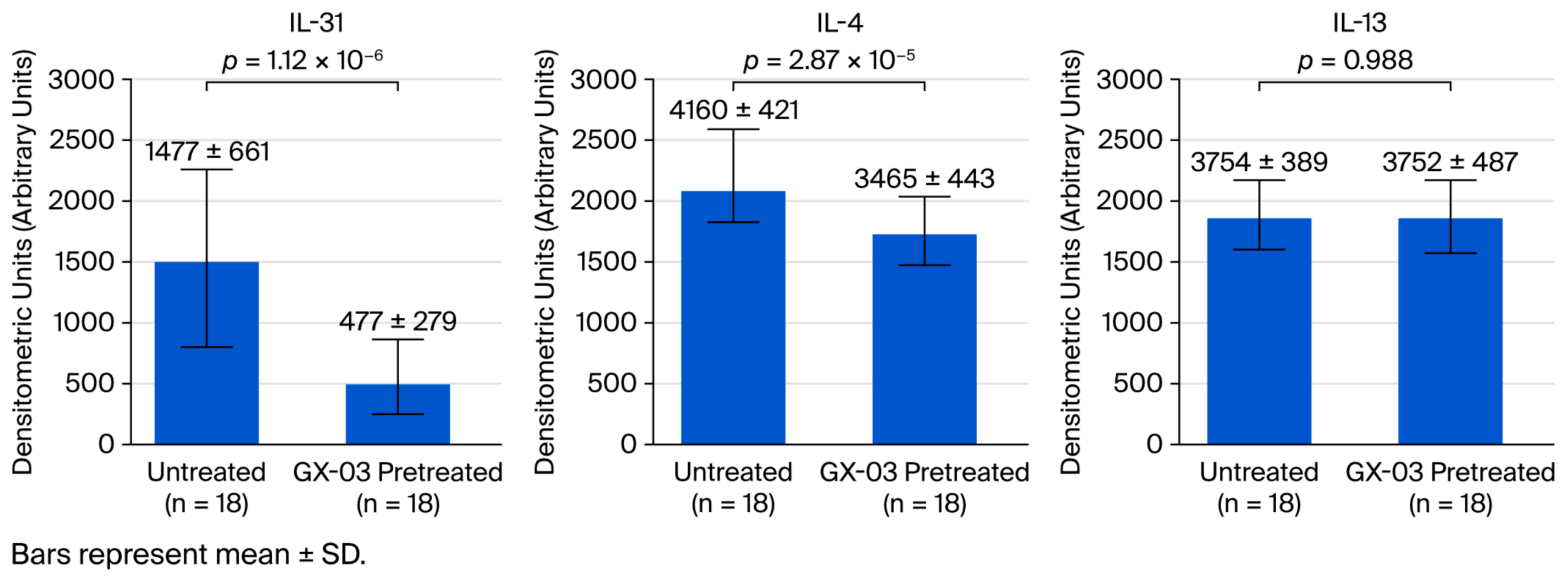
Densitometric Units (AU) for IL-31, IL-4 and IL-13 in Untreated and GX-03 Pretreated animals.

As shown in Figure 5, evaluation of downstream inflammatory cytokines demonstrated selective modulation of the inflammatory response following GX-03 pretreatment (Figure 5). IL-31 demonstrated the largest treatment-associated reduction, decreasing from 1477 ± 661 AU in untreated animals to 477 ± 279 AU following GX-03 pretreatment, representing a 67.7% reduction (exact *p* = 5.12 × 10^−6^) (Figure S3; Table S3). IL-4 expression also demonstrated a significant reduction (Figure S4; Table S4), whereas IL-13 expression did not differ significantly between treatment groups (exact *p* = 0.988) (Figure S5; Table S5). These findings remained unchanged following Holm adjustment.

## 4. Discussion

The present study demonstrates that pretreatment with extended-release polyhexanide formulation GX-03 was associated with attenuation of clinical dermatitis symptoms and reduced expression of several inflammatory cytokines following standardized epicutaneous *Staphylococcus aureus* challenge in a murine model. Specifically, GX-03 pretreatment was associated with marked reductions in IL-36α and IL-36γ expression together with significant decreases in IL-31 and IL-4, whereas IL-13 expression remained unchanged. These findings suggest that modification of the cutaneous microenvironment before standardized bacterial challenge was associated with selective attenuation of epithelial stress-associated and downstream inflammatory cytokine responses.

IL-36 cytokines have emerged as important amplifiers of epithelial inflammatory responses in inflammatory skin disease. Produced primarily by keratinocytes, IL-36α and IL-36γ are rapidly induced following epithelial injury and microbial stimulation and contribute to activation of dendritic cells, neutrophil recruitment, and amplification of downstream inflammatory pathways [13,14]. Increased IL-36 expression has been demonstrated in both human atopic dermatitis and experimental models of cutaneous inflammation, supporting its role as an important component of the early innate immune response [15,16]. In the present study, pretreatment with GX-03 was associated with approximately 50% reductions in both IL-36α and IL-36γ expression following standardized bacterial challenge. However, because bacterial burden, epithelial signaling pathways, and mechanistic endpoints were not evaluated, the present study cannot determine whether these reductions reflect direct effects on inflammatory signaling, indirect effects secondary to altered microbial interactions with the epidermis, or other properties of the complete GX-03 formulation.

Evaluation of downstream cytokines demonstrated differential treatment-associated effects. IL-31 exhibited the largest reduction among the downstream mediators examined, while IL-4 demonstrated a more modest but statistically significant decrease and IL-13 expression remained unchanged. IL-31 has an established role in pruritic signaling and contributes to neuroimmune interactions in inflammatory skin disease, whereas IL-4 is an important mediator of early type 2 immune responses [17]. The absence of a measurable effect on IL-13 may reflect differences in cytokine kinetics during acute inflammation, as IL-13 is often more prominent during chronic disease and tissue remodeling than during the early inflammatory phase represented in this model [18]. Collectively, these findings suggest that GX-03 pretreatment was associated with attenuation of selected inflammatory pathways activated during acute bacterial dermatitis rather than uniform suppression of all measured cytokines.

The mechanism responsible for the observed cytokine reductions cannot be established from the present study. Polyhexanide possesses broad-spectrum antimicrobial activity and may reduce inflammatory stimulation by limiting bacterial interactions with the epidermis. Alternatively, attenuation of cytokine expression may reflect indirect effects related to characteristics of the GX-03 formulation. Because this investigation evaluated the formulation as a whole, the contribution of individual formulation components cannot be determined. Likewise, post-inoculation bacterial burden, antimicrobial peptide expression, and intracellular signaling pathways were not measured, precluding mechanistic conclusions. Future studies incorporating quantitative bacterial cultures, histologic evaluation, epithelial signaling analyses, and component-specific investigations will be important to better define the biological basis for these observations.

Current therapies for atopic dermatitis primarily target inflammatory pathways after disease has become established [19,20]. In contrast, the present study evaluated pretreatment before standardized bacterial challenge and therefore represents a different experimental paradigm. While this approach does not directly model treatment of established disease, it provides insight into how modification of the cutaneous environment before standardized microbial challenge may influence subsequent inflammatory responses. Whether similar cytokine changes occur in established dermatitis or in human disease remains unknown and will require additional clinical investigation.

Although the present investigation was performed in an acute murine model, the findings have potential translational relevance to human atopic dermatitis. *Staphylococcus aureus* colonization is well recognized as a contributor to disease severity and exacerbations in patients with atopic dermatitis through disruption of the skin barrier and activation of inflammatory cytokine pathways, including IL-36 [5]. The association between GX-03 pretreatment and reduced expression of several inflammatory mediators following standardized bacterial challenge raises the possibility that topical strategies capable of modifying the cutaneous inflammatory response may complement existing therapies. However, because this study evaluated prophylactic treatment in an experimental animal model, these findings should be considered hypothesis-generating and cannot be extrapolated directly to established human atopic dermatitis. Clinical studies will be required to determine whether similar biological and clinical effects occur in patients.

This study has several strengths. A standardized *Staphylococcus aureus* inoculation protocol was employed using a verified bacterial concentration, clinical disease severity was evaluated using blinded investigator assessment, and cytokine expression was quantified using standardized Western blot methodology. The consistent reductions observed across multiple inflammatory mediators, together with the largestandardized effect sizes for IL-36α, IL-36γ, and IL-31, support the reproducibility of the observed biological response within this experimental model.

Several limitations should be acknowledged. This investigation evaluated cytokine expression in an acute murine model following pretreatment with the complete proprietary GX-03 formulation and therefore cannot establish causality or define the precise mechanisms responsible for the observed reductions in inflammatory mediators. Histologic changes, post-inoculation bacterial burden, antimicrobial peptide expression, pruritic behavior, epithelial stress signaling, and other downstream immunologic pathways were not evaluated. Because the complete formulation, rather than its individual components, was studied, the relative contribution of polyhexanide and other excipients cannot be determined. Finally, although this model reproduces several features of bacteria-associated cutaneous inflammation, caution is warranted when extrapolating these findings to the complex pathobiology of chronic human atopic dermatitis.

## 5. Conclusions

In summary, pretreatment with extended-release polyhexanide formulation GX-03 significantly reduced clinical dermatitis severity and reduced expression of IL-36α, IL-36γ, IL-31, and IL-4 following a standardized epicutaneous *Staphylococcus aureus* challenge in a murine model, while IL-13 expression remained unchanged. These findings demonstrate selective attenuation of epithelial stress-associated and downstream inflammatory cytokine responses following bacterial challenge in this experimental model. Further studies are warranted to define the biological mechanisms responsible for these observations and to better characterize the relationship between GX-03 pretreatment, microbial challenge, and inflammatory cytokine signaling.

## Figures and Tables

**Table 1:** Densitometric analysis of inflammatory cytokine expression in untreated and GX-03-pretreated mice.

Cytokine	Untreated Mean ± SD	GX-03 Mean ± SD	% Change	Exact *p*
IL-36α	5586 ± 701	2788 ± 761	−49.9	3.08 × 10^−13^
IL-36γ	4865 + 401	2479 +1387	−50.9	8.88 × 10^−7^
IL-31	1477 ± 661	477 ± 279	−67.7	5.12 × 10^−6^
IL-4	4160 ± 421	3465 ± 443	−16.7	2.87 × 10^−5^
IL-13	3754 ± 389	3752 + 487	0.0	0.988

Values are presented as mean ± SD (*n* = 18 animals/group). Exact *p* values represent the primary statistical comparison. Holm-adjusted *p* values, 95% confidence intervals, effect sizes (Cohen’s d), and statistical test selection are reported in the Results section.

## Data Availability

All data presented in this study are publicly available at: https://doi.org/10.6084/m9.figshare.31938768.

## Supplementary Materials

The following supporting information can be downloaded at: https://www.mdpi.com/article/10.3390/life16081369/s1, Figure S1: (A) Representative Western blot of IL-36α (predicted molecular weight 20 kDa) in skin lysates from Group 1 (untreated) and Group 2 (GX-03-pretreated) mice following *S. aureus* exposure. Lanes are ordered by animal number, Animal #1–18 (*n* = 18/group). (B) Lane-by-lane densitometric analysis: the paired blot scan (Group 1 band directly above Group 2 band per animal, Lane #1–18) as analyzed in GelAnalyzer 23.1.1 to generate the raw integrated density values in Table S1. (C) Quantification of band intensity (mean ± SD, raw integrated density) for Group 1 vs. Group 2; two-tailed *t*-test *p* = 3.08 × 10^−13^; Figure S2: (A) Representative Western blot of IL-36γ (predicted molecular weight 19 kDa) in skin lysates from Group 1 (untreated) and Group 2 (GX-03-pretreated) mice following *S. aureus* exposure. Lanes are ordered by animal number, Animal #1–18 (*n* = 18/group). (B) Lane-by-lane densitometric analysis: the paired blot scan (Group 1 band directly above Group 2 band per animal, Lane #1–18) as analyzed in GelAnalyzer 23.1.1 to generate the raw integrated density values in Table S2. (C) Quantification of band intensity (mean ± SD, raw integrated density) for Group 1 vs. Group 2; two-tailed *t*-test *p* = 4.35 × 10^−8^; Figure S3: (A) Representative Western blot of IL-31 (predicted molecular weight 16 kDa) in skin lysates from Group 1 (untreated) and Group 2 (GX-03-pretreated) mice following *S. aureus* exposure. Lanes are ordered by animal number, Animal #1–18 (*n* = 18/group). (B) Lane-by-lane densitometric analysis: the paired blot scan (Group 1 band directly above Group 2 band per animal, Lane #1–18) as analyzed in GelAnalyzer 23.1.1 to generate the raw integrated density values in Table S3. (C) Quantification of band intensity (mean ± SD, raw integrated density) for Group 1 vs. Group 2; two-tailed *t*-test *p* = 1.12 × 10^−6^; Figure S4: (A) Representative Western blot of IL-4 (predicted molecular weight 14 kDa) in skin lysates from Group 1 (untreated) and Group 2 (GX-03-pretreated) mice following *S. aureus* exposure. Lanes are ordered by animal number, Animal #1–18 (*n* = 18/group). (B) Lane-by-lane densitometric analysis: the paired blot scan (Group 1 band directly above Group 2 band per animal, Lane #1–18) as analyzed in GelAnalyzer 23.1.1 to generate the raw integrated density values in Table S4. (C) Quantification of band intensity (mean ± SD, raw integrated density) for Group 1 vs. Group 2; two-tailed *t*-test *p* = 2.87 × 10^−5^; Figure S5: (A) Representative Western blot of IL-13 (predicted molecular weight 12 kDa) in skin lysates from Group 1 (untreated) and Group 2 (GX-03-pretreated) mice following *S. aureus* exposure. Lanes are ordered by animal number, Animal #1–18 (*n* = 18/group). (B) Lane-by-lane densitometric analysis: the paired blot scan (Group 1 band directly above Group 2 band per animal, Lane #1–18) as analyzed in GelAnalyzer 23.1.1 to generate the raw integrated density values in Table S5. (C) Quantification of band intensity (mean ± SD, raw integrated density) for Group 1 vs. Group 2; two-tailed *t*-test *p* = 0.988, not significant; Table S1: Raw integrated density values (arbitrary units, GelAnalyzer 23.1.1) for the IL-36α Western blot shown in Figure S1. Values correspond to Group 1 (untreated) and Group 2 (GX-03-pretreated) lanes by animal number; Table S2: Raw integrated density values (arbitrary units, GelAnalyzer 23.1.1) for the IL-36γ Western blot shown in Figure S2. Values correspond to Group 1 (untreated) and Group 2 (GX-03-pretreated) lanes by animal number; Table S3: Raw integrated density values (arbitrary units, GelAnalyzer 23.1.1) for the IL-31 Western blot shown in Figure S3. Values correspond to Group 1 (untreated) and Group 2 (GX-03-pretreated) lanes by animal number; Table S4: Raw integrated density values (arbitrary units, GelAnalyzer 23.1.1) for the IL-4 Western blot shown in Figure S4. Values correspond to Group 1 (untreated) and Group 2 (GX-03-pretreated) lanes by animal number; Table S5: Raw integrated density values (arbitrary units, GelAnalyzer 23.1.1) for the IL-13 Western blot shown in Figure S5. Values correspond to Group 1 (untreated) and Group 2 (GX-03-pretreated) lanes by animal number.
