# Supplementary material for: Effects of Extended-Release Polyhexanide on Inflammatory Cytokine Expression and Disease Severity Following Staphylococcus Aureus Exposure in a Murine Dermatitis Model
Source: Life (Basel). 2026 Aug 20;16(8):1369. doi: 10.3390/life16081369 (PMC13514718; doi:10.3390/life16081369)
Supplement: Supplementary file 1 [file life-16-01369-s001.zip › life-4434006-supplementary.pdf]

Supplementary Data

IL-36 alpha

(A) Western Blot Target-Band Images for All Biological Replicates

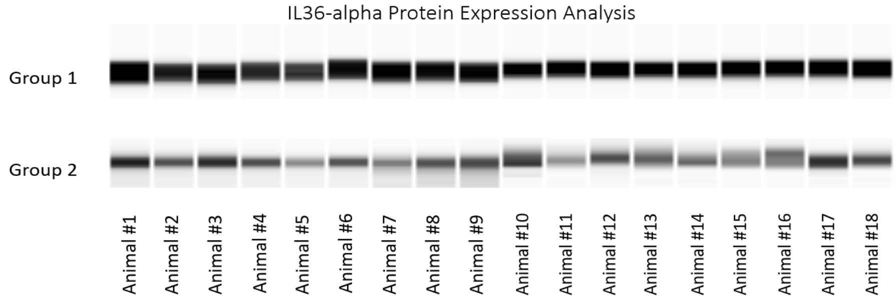

(B) Densitometric Analysis

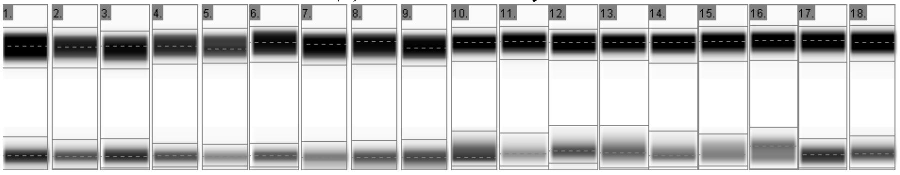

(C) Quantification (Mean ± SD)

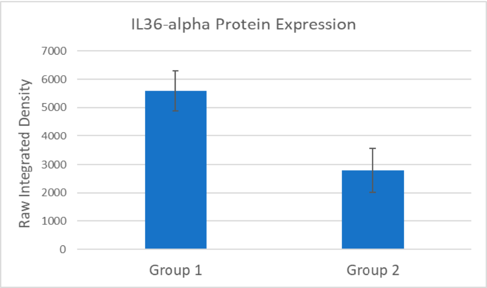

Figure S1. (A) Representative Western blot of IL-36α (predicted molecular weight 20 kDa) in skin lysates from Group 1 (untreated) and Group 2 (GX-03-pretreated) mice following S. aureus exposure. Lanes are ordered by animal number, Animal #1–18 (n = 18/group). (B) Lane-by-lane densitometric analysis: the paired blot scan (Group 1 band directly above Group 2 band per animal, Lane #1–18) as analyzed in GelAnalyzer 23.1.1 to generate the raw integrated density values in Table S1. (C) Quantification of band intensity (mean ± SD, raw integrated density) for Group 1 vs. Group 2; two-tailed t-test $p = 3.08 \times 10^{-13}$.

Animal #	Group 1	Group 2
1	7603	3675
2	5517	2417
3	5701	3109
4	5099	2225
5	4729	1330
6	5650	2052
7	6515	1981
8	5958	2861
9	6180	3272
10	5208	3886
11	5085	1588
12	5156	3365
13	4821	3654
14	5153	2435
15	5256	2382
16	5258	3473
17	5481	3409
18	6176	3067

Table S1. Raw integrated density values (arbitrary units, GelAnalyzer 23.1.1) for the IL-36α Western blot shown in Figure S1. Values correspond to Group 1 (untreated) and Group 2 (GX-03-pretreated) lanes by animal number.

IL-36 gamma

(A) Representative Western Blot Band Images

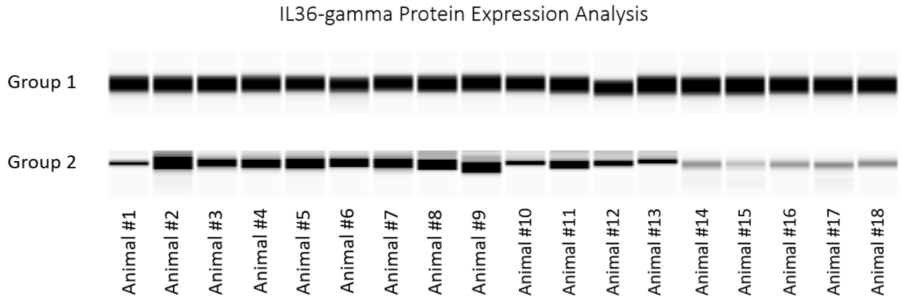

(B) Densitometric Analysis

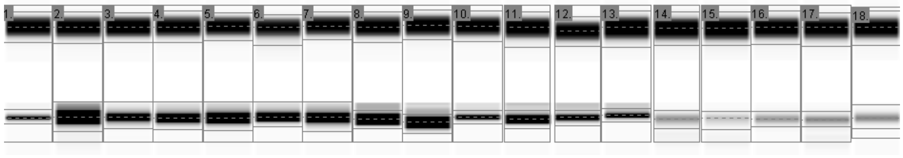

(C) Quantification (Mean ± SD)

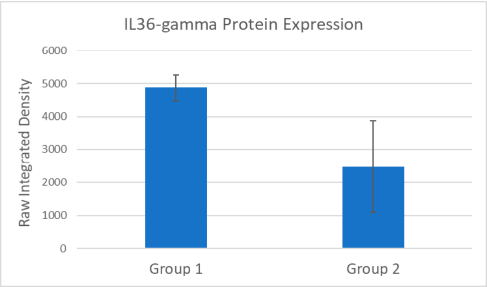

Figure S2. (A) Representative Western blot of IL-36γ (predicted molecular weight 19 kDa) in skin lysates from Group 1 (untreated) and Group 2 (GX-03-pretreated) mice following S. aureus exposure. Lanes are ordered by animal number, Animal #1–18 (n = 18/group). (B) Lane-by-lane densitometric analysis: the paired blot scan (Group 1 band directly above Group 2 band per animal, Lane #1–18) as analyzed in GelAnalyzer 23.1.1 to generate the raw integrated density values in Table S2. (C) Quantification of band intensity (mean ± SD, raw integrated density) for Group 1 vs. Group 2; two-tailed t-test $p = 4.35 \times 10^{-8}$.

Animal #	Group 1	Group 2
1	4818	1313
2	4765	4982
3	4790	3126
4	4710	3581
5	4343	4026
6	4072	3391
7	4375	4025
8	4752	3524
9	4780	3687
10	4593	1695
11	5349	3133
12	4716	2184
13	5498	1785
14	5564	950
15	5312	543
16	4992	753
17	5000	841
18	5139	1089

Table S2. Raw integrated density values (arbitrary units, GelAnalyzer 23.1.1) for the IL-36γ Western blot shown in Figure S2. Values correspond to Group 1 (untreated) and Group 2 (GX-03-pretreated) lanes by animal number.

IL-31

(A) Representative Western Blot Band Images

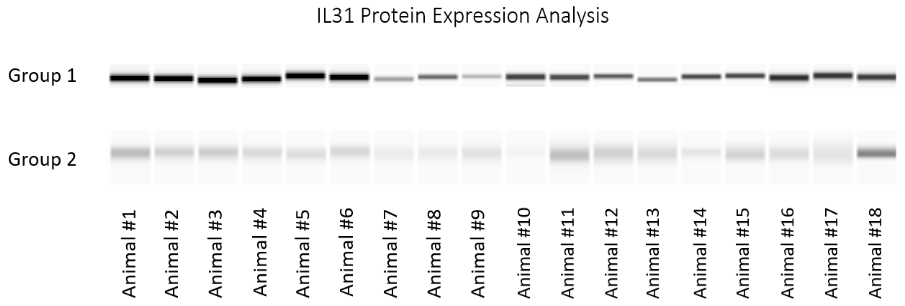

(B) Densitometric Analysis

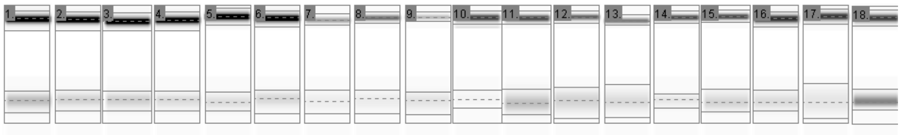

(C) Quantification (Mean ± SD)

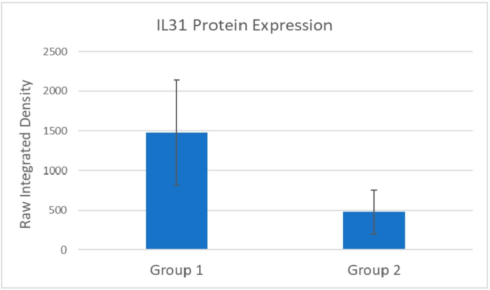

Figure S3. (A) Representative Western blot of IL-31 (predicted molecular weight 16 kDa) in skin lysates from Group 1 (untreated) and Group 2 (GX-03-pretreated) mice following S. aureus exposure. Lanes are ordered by animal number, Animal #1–18 (n = 18/group). (B) Lane-by-lane densitometric analysis: the paired blot scan (Group 1 band directly above Group 2 band per animal, Lane #1–18) as analyzed in GelAnalyzer 23.1.1 to generate the raw integrated density values in Table S3. (C) Quantification of band intensity (mean ± SD, raw integrated density) for Group 1 vs. Group 2; two-tailed t-test $p = 1.12 \times 10^{-6}$.

Animal #	Group 1	Group 2
1	2353	751
2	2323	515
3	2157	515
4	2229	396
5	2257	359
6	2227	510
7	543	233
8	877	253
9	370	257
10	1216	74
11	1242	767
12	965	730
13	727	555
14	1121	227
15	1095	462
16	1687	369
17	1633	328
18	1572	1284

Table S3. Raw integrated density values (arbitrary units, GelAnalyzer 23.1.1) for the IL-31 Western blot shown in Figure S3. Values correspond to Group 1 (untreated) and Group 2 (GX-03-pretreated) lanes by animal number.

IL-4

(A) Representative Western Blot Band Images

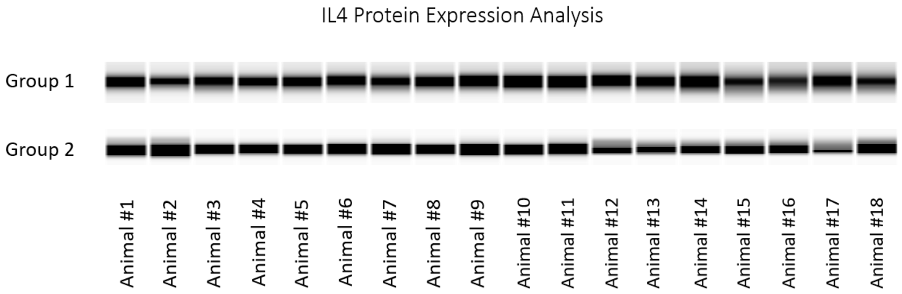

(B) Densitometric Analysis

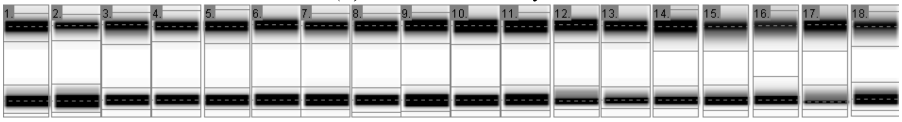

(C) Quantification (Mean ± SD)

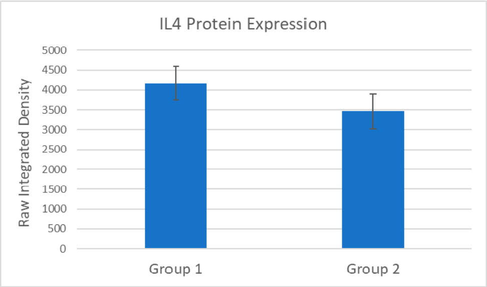

Figure S4. (A) Representative Western blot of IL-4 (predicted molecular weight 14 kDa) in skin lysates from Group 1 (untreated) and Group 2 (GX-03-pretreated) mice following S. aureus exposure. Lanes are ordered by animal number, Animal #1–18 (n = 18/group). (B) Lane-by-lane densitometric analysis: the paired blot scan (Group 1 band directly above Group 2 band per animal, Lane #1–18) as analyzed in GelAnalyzer 23.1.1 to generate the raw integrated density values in Table S4. (C) Quantification of band intensity (mean ± SD, raw integrated density) for Group 1 vs. Group 2; two-tailed t-test $p = 2.87 \times 10^{-5}$.

Animal #	Group 1	Group 2
1	4468	4109
2	3258	4396
3	4043	3215
4	3709	3099
5	4374	3445
6	4308	3502
7	3793	3499
8	4168	3202
9	4027	3865
10	4605	3461
11	4277	3630
12	4534	3301
13	4023	2762
14	4707	3190
15	4455	3552
16	3721	3621
17	4808	2592
18	3608	3921

Table S4. Raw integrated density values (arbitrary units, GelAnalyzer 23.1.1) for the IL-4 Western blot shown in Figure S4. Values correspond to Group 1 (untreated) and Group 2 (GX-03-pretreated) lanes by animal number.

IL-13

(A) Representative Western Blot Band Images

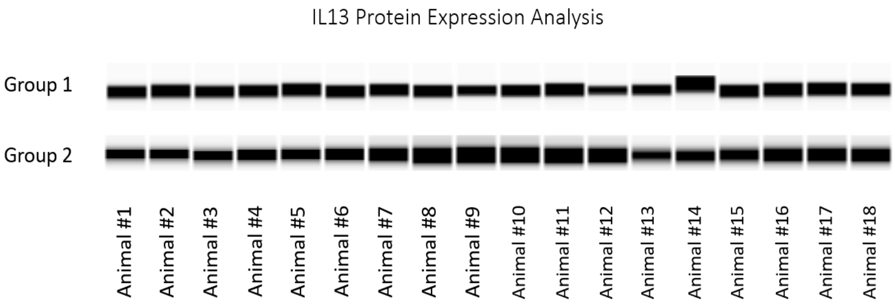

(B) Densitometric Analysis

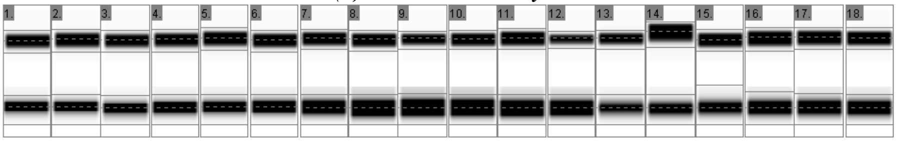

(C) Quantification (Mean ± SD)

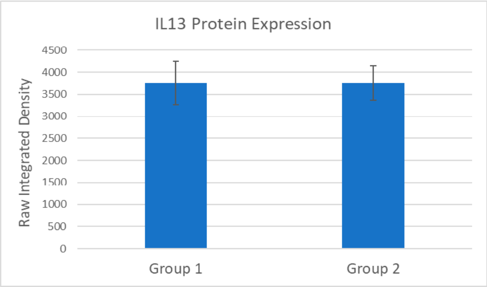

Figure S5. (A) Representative Western blot of IL-13 (predicted molecular weight 12 kDa) in skin lysates from Group 1 (untreated) and Group 2 (GX-03-pretreated) mice following S. aureus exposure. Lanes are ordered by animal number, Animal #1–18 (n = 18/group). (B) Lane-by-lane densitometric analysis: the paired blot scan (Group 1 band directly above Group 2 band per animal, Lane #1–18) as analyzed in GelAnalyzer 23.1.1 to generate the raw integrated density values in Table S5. (C) Quantification of band intensity (mean ± SD, raw integrated density) for Group 1 vs. Group 2; two-tailed t-test p = 0.988, not significant.

Animal #	Group 1	Group 2
1	3773	3376
2	3908	3167
3	3561	3263
4	3851	3729
5	4055	3494
6	3843	3782
7	3783	3997
8	3701	4123
9	3032	4134
10	3517	4179
11	3883	4208
12	2578	4293
13	3113	3053
14	4831	3420
15	3962	3576
16	4110	3836
17	3990	3857
18	4038	4082

Table S5. Raw integrated density values (arbitrary units, GelAnalyzer 23.1.1) for the IL-13 Western blot shown in Figure S5. Values correspond to Group 1 (untreated) and Group 2 (GX-03-pretreated) lanes by animal number.
